# Progress towards universal health coverage in the context of mental disorders in India: evidence from national sample survey data

**DOI:** 10.1186/s13033-023-00595-6

**Published:** 2023-09-19

**Authors:** Alok Ranjan, Jewel E Crasta

**Affiliations:** 1 School of Liberal Arts, Centre for Emerging Technology and Sustainable Development, Indian Institute of Technology, Jodhpur, India; 2 Department of Global Health and Population, Harvard T. H. Chan School of Public Health, Boston, USA; 3 School of Health and Rehabilitation Sciences, The Ohio State University, Columbus, USA

**Keywords:** Mental health, India, National Sample Survey, Economics, Out-of-pocket expenditure, Universal Health Coverage

## Abstract

**Background:**

Universal health coverage (UHC) has emerged as one of the important health policy discourses under the current sustainable development goals in the world. UHC in individual disease conditions is a must for attaining overall UHC. This study measures progress towards UHC in terms of access to health care and financial protection among individuals with mental disorders in India.

**Methods:**

Data from the 75th Round National Sample Survey (NSS), 2017-18, was used, which is the latest round on health in India. Data collected from 555,115 individuals (rural: 325,232; urban: 229,232), from randomly selected 8077 villages and 6181 urban areas, included 283 outpatient and 374 hospitalization cases due to mental disorders in India. Logistic regression models were used for analyses.

**Results:**

Self-reporting of mental disorders was considerably lower than the actual disease burden in India. However, self-reporting of ailment was 1.73 times higher (95% CI: 1.18–2.52, p < 0.05) among the richest income group population compared to the poorest in India. The private sector was a major service provider of mental health services with a larger share for outpatient (66.1%) than inpatient care (59.2%). Over 63% of individuals with a mental disorder who reported private sector hospitalization noted unavailability or poor service quality at public facilities. Only 23% of individuals hospitalized had health insurance coverage at All India level. However, health insurance coverage among poorest economic class was a meagre 3.4%. Average out-of-pocket expenditure during hospitalization (public: 123 USD; private: 576 USD) and outpatient care (public: 8 USD; private: 37 USD) was significantly higher in the private sector than in the public sector. Chances of facing catastrophic health expenditure at 10% threshold were 23.33 times (95% CI: 10.85–50.17; p < 0.001) higher under private sector than public sector during hospitalization. Expenditure on medicine, as the share of total medical expenditure, was highest for hospitalization (public: 45%, private:39.5%) and outpatient care (public: 74.1%, private:39.7%).

**Conclusions:**

Social determinants play a vital role in access to healthcare and financial protection among individuals with mental disorders in India. For achieving UHC in mental disorders, India needs to address the gaps in access and financial protection for individuals with mental disorders.

**Trial registration:**

Not applicable.

**Supplementary Information:**

The online version contains supplementary material available at 10.1186/s13033-023-00595-6.

## Background

Universal Health Coverage (UHC) is a significant policy discourse globally. It is also Goal-3 under Sustainable Development Goals (SDGs). UHC is defined as the desired outcome of the health system whereby all individuals who need health services (promotion, prevention, treatment, rehabilitation, and palliation) can receive them without facing financial hardships [1, 2]. Access and financial protection are fundamental aspects of UHC. Achieving this goal, therefore, requires UHC for each disease condition independently [3]. Considering the high burden of mental disorders in the world and India, this study aims to explore where India is, with respect to achieving UHC for mental disorders and the reasons behind the persisting gaps.

The 2017 National Mental Health Survey indicated that around 197.3 million individuals had a mental disorder in India [4]. In 2016, India accounted for over 26% of worldwide suicide-related deaths [5]. These pre-COVID-19 statistics reveal that morbidity and mortality associated with mental disorders in India were already high. Emerging research has shown that the COVID-19 pandemic has resulted in a sharp rise in mental health concerns [6], further highlighting that mental disorders are a public health priority. There is an urgent need for research examining social determinants of health and patterns of healthcare access for individuals with mental disorders.

Social determinants of healthcare utilization (an indicator of access) among individuals with mental disorders include age, sex, race, socioeconomic status, education level, marital status, and rural and urban settings [7–9]. Social determinants are non-medical factors in which people are born, grow, work, live and wider sets of forces that shape the daily life. These determinants have an unfair and avoidable impact on health outcomes of individuals and society [10]. Research show that social determinants have a larger impact on health than healthcare and lifestyle choices. Various studies show that social determinants account for 30–55% of health outcomes [10].

A study investigating the social correlates of mental, neurological, and substance use disorders in India and China showed that social factors differentially impacted prevalence in India compared to higher-income countries [11]. Specifically, there was a positive association between being married and depression among women in India, and low education and poverty were associated with a higher occurrence of dementia [11]. Rural areas within India reported a higher prevalence (17.1/1000) of mental disorders compared to urban areas (12.7/1000); [9]. Disparities in economic profiles across rural and urban regions, further contribute to inequities in healthcare access. Additionally, expenditure incurred for mental health services is alarmingly high, often leading families to an economic crisis [12]. An estimated 85% of individuals with mental disorders seek treatment from the private sector [13], and private-sector out-of-pocket expenditure (OOPE) is nearly five times higher than that in the public sector. OOPE incurred by individuals with mental disorders includes medicines, psychiatrist fees, travel, as well as losing wages on the day of visiting the doctor [12]. Catastrophic health expenditure, defined as expenditure exceeding 10% of household expenses (CHE-10), was incurred by around 63% of individuals with psychiatric or neurological disorders and was significantly higher among the poorest quintile [14]. The direct and indirect costs of mental disorders can worsen the economic condition, creating a vicious cycle of poverty and mental disorders [8]. These findings emphasize the importance of understanding the local sociodemographic context while designing strategies to reduce the disease burden of mental disorders. Given the socio-cultural and demographic diversity across regions in India, a systematic investigation into these factors is required.

The current study evaluated data from individuals with mental disorders from the most recently released (November 2019) 75th Round National Sample Survey (75th NSS). In the 75th NSS, mental disorders include psychiatric disorders, operationally defined as diseases of longer duration of irregular nature affecting behavior/ abnormal behavior including excessive fears, anger, and violence; depression; detached from reality and drug abuse or alcoholism, defined as drug abuse or alcoholism interfering with the performance of major life activities such as learning, thinking, communicating, sleeping, etc. Based on available data in NSS 75th Round, healthcare utilization has been taken as an access indicator for this study. Based on prior research showing relationships between demographic characteristics and healthcare utilization among individuals with mental disorders, we investigated the impact of age, place of residence, gender, marital status, social group classification, education, employment level, and socioeconomic status on healthcare utilization and financial protection, which are core dimensions of UHC. To the best of our knowledge this is one the few studies which have explored access and financial protection among individuals with mental disorders at All India level.

## Methods

The present study used an anonymized secondary level unit data set of the 75th NSS, conducted from July 2017 to June 2018, made available in the public domain for research purposes by the Ministry of Statistics and Programme Implementation, Government of India. This nationwide survey was conducted by the Government of India. The NSS was set up by Government of India in 1950 to collect various aspects of data, including health, on a periodic interval. Methodology of NSS is approved by an expert group at the national level. The 75th NSS measures social consumption of health in India. The survey’s unit-level data and report of the survey were released in November 2019, making it one of the latest unit-level national data sets available in the country [15]. This survey covered all 30 States and six Union Territories of India except for those villages of Andaman and Nicobar which are inaccessible. Survey followed a stratified multi-stage sampling, where the sampling frame for the rural areas was the list of 2011 Census villages, and the sampling frame for urban areas was Urban Frame Survey blocks (UFS 2007-12). Within each district of the state and union territories, rural and urban strata were formed. Total 14,300 first sampling units (FSUs) were allotted for the central sample and sample FSUs were allocated to States and union territories in proportion to population based on census 2011 subject to minimum sample allocated to each state. Households listed under FSUs or sub-FSUs were further stratified under second stage strata and from second state strata household sample was taken. The 75th NSS collected data from 5, 55,115 individuals (rural: 3, 25,883; urban: 2, 29,232; male: 2,83,200; female: 2,71,877) and 1,13,823 households from randomly selected 8,077 rural villages and 6,181 urban blocks. Sample size at the state level was in proportion to the state’s population size. For instance, sample size for the Uttar Pradesh State (India’s most populous state) was 61,904 (highest in all sample) and for Goa (one of the smallest states) was 2036 [15]. Socioeconomic sample characteristics was representative of India’s population. For example, weighted sample of Scheduled Tribe (ST-9.07%), Scheduled Caste (SC-19.63%), Other Backward Caste (OBC-44.92%) in 75th NSS triangulate well with Government of India’s Socio Economic Caste Census, 2011 (ST-8.5%, SC-19.7%, and OBC-41.1%) [16].

For a study on mental health conditions, it is important to compare the sample of 75th NSS, 2017-18, with the National Mental Health Survey (NMHS) 2015-17 for assessing the representativeness of the sample. The NMHS is in-depth exploration of health system issues and healthcare needs of persons with mental disorder. Both the 75th NSS and NMHS were commenced by Government of India employing scientific sampling methods (multi-stage, stratified, random sampling). However, the 75th NSS sample represented all 30 States and six Union Territories in India, whereas the NMHS sample represented 12 states across 6 regions [North (Punjab and Uttar Pradesh); South (Tamil Nadu and Kerala); East (Jharkhand and West Bengal); West (Rajasthan and Gujarat); Central (Madhya Pradesh and Chhattisgarh) and North-east (Assam and Manipur)]. The 75th NSS has a total sample of 5,55,115 individuals out of which 283 outpatient and 374 hospitalization cases were reported due to mental disorders, whereas the NMHS has a smaller sample of 39,532 individuals. The 75th NSS used one liner self-reporting of mental disorders by quantitative methods, whereas NMHS used 10 instruments including Mini International Neuro-psychiatric Interview through quantitative and qualitative methods. Self-reporting estimates of 75th NSS is reliable at the all India level but not at the state level due to low sample size at the state level, whereas NMHS estimates at the state level are reliable and robust with an adequate sample size.

The 75th NSS data were collected through self-report from members of the household and included sociodemographic profile, nature of ailments, morbidity in the last 15 days, hospitalization and mortality in the last 365 days, elderly health, immunization coverage, maternal health and childbirth, insurance coverage, out-of-pocket expenditure (OOPE), and choice of healthcare provider. Mental disorders are one of the 63 ailment categories in the survey.

### Demographic and socioeconomic variables

In some analyses variables and variable categories were retained as provided by 75th NSS whereas in certain situations they was re-categorized based on study’s objectives. Details of the variables and their categorization are as follows:

Age was a numeric variable in the 75th NSS data. For analyses, it was categorized under the broader categories of ‘0–14’, ’15–29’, ‘30–44’, ‘45–59’, and ‘60+’ years.

Marital status data was collected as never married, currently married, widowed, divorced, or separated. In our analysis, never married, divorced, or separated were grouped together.

Social groups, also called *caste* in India, were categorized as scheduled tribe (ST), scheduled caste (SC), other backward castes (OBCs), and general. These categories are constitutional categories and were provided in the 75th NSS data. STs refer to India’s indigenous population in tribal communities and are classified according to their unique cultural customs and geographical seclusion, rather than their position within the caste system [17]. SCs refer to individuals at the bottom of the hierarchical caste system and have historically faced social, occupational, and educational discrimination and oppression. The criteria for categorizing a caste or community as an OBC include factors such as social and educational backwardness, lack of access to resources and opportunities, and historical disadvantage. The general category consists of the rest of the castes that do not fit the SC, ST, and OBC categories, and include castes that occupy the top of the traditional caste system hierarchy. According to the 2011 Census of India, SCs and STs together make up approximately 25% of the country’s population [18].

Education level data were collected by 75th NSS as follows: not literate, literate without any schooling, literate without formal schooling: through National Fundamental Education Centre (NFEC), literate through Total Literacy Campaign (TLC)/ Adult Education Centres (AEC), others; literate with formal schooling: below primary, primary, upper primary/middle, secondary, higher secondary, diploma /certificate course (upto secondary), diploma/certificate course (higher secondary), diploma/certificate course (graduation & above), graduate, post graduate and above. For analyses, it was re-categorized as illiterate, up to the primary, up to secondary, and above secondary.

Household occupation was categorized as self-employed, regular wages, and casual laborer. Economic quintiles were created from reports of usual monthly per capita consumption expenditure (UMPCE) for households. Based on the UMPCE, usual annual per capita consumption expenditure (UAPCE) was calculated and the following five economic class were generated: poorest, poor, middle, rich, and richest. Similar methodologies were also followed in previous studies of similar datasets [19–21].

Proportion of the ailing population (PAP) was calculated per 1,00,000 population if any household member reported any acute or chronic ailment in the last 15 days.

Hospitalization rate was calculated based on the proportion of individuals who reported an incidence of hospitalization due to mental disorders in the last 365 days.

Public and private sector use for outpatient care and hospitalization was based on an individual’s choice of healthcare provider which included health sub-centre (HSC), primary health centre (PHC), community health centre (CHC), district hospital (DH), government medical colleges, charitable or trust run hospital, private hospital, private doctor or clinic, and informal healthcare provider. In this analysis, HSC, PHC, CHC, DH, and government medical colleges were re-categorized as public providers. In contrast, private hospitals, private clinics, charitable or trust-run hospitals, and informal providers were re-categorized as private providers.

Health insurance coverage categories included: (1) Government-sponsored (example –RSBY, PMJAY, Arogyasri, etc.), (2) Government /PSU as an employer (example-CGHS, reimbursement from government, etc.), (3) Employer-supported (example: ESIS), (4) Private insurance and (5) Not covered at all. This variable was provided by 75th NSS and it was retained as it is during the analysis.

Expenditure for medical and non-medical needs was based on detailed expense reports for outpatient visits in the last 15 days or hospitalization in the last 365 days. Medical expenditure included doctor’s fees, medicine, diagnostic test, and other medical expenses, whereas non-medical expenditure included transportation and other non-medical expense.

Out-of-pocket expenditure (OOPE) variable was calculated by adding total medical expenditure and transportation, followed by subtracting reimbursement by insurance companies or employers.

Catastrophic health expenditure at 10% (CHE-10) was defined as OOPE higher than 10% of the usual annual per capita consumption expenditure [19, 22]. This variable was calculated from the unit level record of 75th NSS.

Sources of financing for total medical expenditure were categorized by 75th NSS as household income or savings, borrowing, sale of the physical asset, a contribution from friends and relatives, and other sources. It was retained as is for the analysis.

Barriers to availing public healthcare facilities were collected from individuals who did not utilize public healthcare facilities and included: services not available, available but poor quality or doctor not available, quality satisfactory but health facility being too far, quality satisfactory but long waiting time, financial constraints, and preference for trusted doctor or hospital. This categorization was given by 75th NSS and it was retained as is for the analysis.

Utilization rate was calculated as a major indicator for access to healthcare during hospitalization and outpatient care. Utilization rate was categorized as public and private sector utilization for the analysis. Public utilization rate included HSC, PHC, CHC, DH, and government medical colleges, whereas as private utilization rate included private hospitals, private clinics, charitable or trust-run hospitals, and informal providers.

### Statistical analyses

Descriptive statistics and logistic regression were used for this study. Descriptions of health-related expenditure are reported in Indian rupees (INR) and United States Dollars (USD) with an exchange rate of 1 USD = 64.45 INR. This exchange rate is the annual average from financial year (FY) 2017-18 [23]. FY 2017-18 was taken as the reference year for the exchange rate since 75th NSS data collection occurred in the same period. To understand the predictors of access and hospitalization three logistic regression models were constructed as follows:

1. Model-1: This model looks at the predictors of access to healthcare for the individuals with mental disorders. The dependent variable was the incidence of hospitalization due to mental disorders in the last 365 days. The explanatory variables (independent variables) included age category, residence, gender, social group, household occupation, income quintile, and insurance coverage.
2. Model-2: Like Model-1 this model also looks at the predictors of access to healthcare for the individuals with mental disorders. The dependent variable was the incidence of reporting acute or chronic ailment in the last 15 days due to mental disorders, referred to as proportion of ailing population (PAP). The explanatory variables were residence, gender, social group, household occupation, and income quintile.
3. Model-3: This model looks at the predictors of financial protection for the individuals with mental disorders. The dependent variable was the incidence of CHE-10 during hospitalization related to mental disorders, and the explanatory variables were age category, residence, gender, social group, education, marital status, household occupation, income quintile, insurance coverage, and type of provider.

Variation inflation factor for multicollinearity, LR-Chi2 and its *p*-value for the goodness of fit, mean pregibon dbeta values for influential observation, and predicted value (_hat)[p>|z|] and predicted value squared (_hatsq) [p>|z|] for specification error were calculated for these models. Literature review and test for specification error helped in identifying the relevant explanatory variable for the models. Weights (multipliers) were provided by NSS in the unit-level data. All analyses were done after applying analytical weight and cross-checked with the 75th NSS report [15]. Similar methods were also used in previous studies [19, 20]. STATA version 14.1 was used for the analyses.

## Results

### Demographic and socioeconomic characteristics of sample population with mental disorders

Out of a total of 93,925 hospitalizations in the last 365 days in India, 2017-18, 374 were due to mental disorders (Table 1). The average age of individuals with a mental disorder and who had a hospitalization in the last 365 days was 38.4 years. Out of a total of 43,240 outpatient visits in the last 15 days, 283 were due to mental disorders. The survey collected gender data as male, female, and transgender. However, in this survey, no transgender individuals reported having a mental disorder.

**Table 1:** Demographic and socioeconomic characteristics of sample population with mental disorders in India, 2017-18

	Mental Disorder			
	Outpatient (283)		Hospitalization (374)	
	Sample size (N)	Percentage	Sample size (N)	Percentage
Total	283 out of total 43,240 outpatient episodes	0.49%	374 out of total 93,925 hospitalization episodes	0.40%
Mean age (years)	41.9		38.4	
Rate of hospitalization and PAP	40 PAP per 1,00,000 population		20 hospitalization per 1,00,000 population	
Rural	30 PAP per 1,00,000 population		20 hospitalization per 1,00,000 population	
Urban	20 PAP per 1,00,000 population		20 hospitalization per 1,00,000 population	
Age group (years)				
0–14	22	11.5	37	21.6
15–29	56	14.1	91	21.7
30–44	80	36.3	99	24.1
45–59	63	17.4	89	23.0
60+	62	20.6	58	9.7
Place of Residence				
Rural	161	60.2	221	73.4
Urban	122	39.8	153	26.6
Gender				
Male	157	59.7	234	57.3
Female	126	40.3	140	42.7
Marital Status				
Never Married/ divorced/ separated	100	42.6	133	44.9
Currently married	154	48.4	203	49.6
Widowed	29	9.0	24	5.5
Social Groups				
ST	11	4.2	24	3.3
SC	39	17.2	63	14.2
OBC	123	40.3	158	55.3
General	110	38.4	129	27.2
Education				
Illiterate	101	33.5	121	33.9
Up to primary	59	28.4	56	23.6
Up to secondary	83	16.5	119	27.9
Above Secondary	40	21.7	64	14.6
Household occupation				
Self employed	143	40.0	177	49.2
Regular Wages	66	18.3	91	25.3
Casual Labourer	44	21.7	62	17.2
Others	30	19.9	30	8.3
Economic quintile				
Poorest	53	17.7	78	24.7
Poor	44	18.0	74	13.5
Middle	61	22.2	67	18.8
Rich	53	17.9	74	23.7
Richest	72	24.2	81	19.3

Source: Authors’ computation from unit records of NSSO 75th Round 2017-18

### Access: Healthcare utilization during hospitalization and outpatient care in India

#### Hospitalization

Out of the total hospitalizations due to mental disorders, 40.8% were under public providers and 59.2% were under private providers (Table 2). Share of public facilities utilization was lowest (21.0%) in the 0–14 years’ age group and highest (59.6%) in the 15–29 years age group. Public facilities utilization was also high among the ST category (75.5%), illiterate (52.0%), self-employed (41.8%), and rich income quintile (49.3%) compared to their respective counterparts. On the other hand, private facility utilization was high among 0–14 years, rural areas, females, OBC category, primary school educated, casual laborer, and poor income quintile.

**Table 2:** Healthcare utilization among individuals with mental disorders during hospitalization and outpatient care in India

	Hospitalization (n = 374)		Out-patient care (n = 283)		
	Public (%)	Private (%)		Public (%)	Private (%)
Total	40.8	59.2		33.9	66.1
Age group (years)					
0–14	21.0	79.0		53.1	46.9
15–29	59.6	40.4		28.2	71.8
30–44	28.4	71.6		34.3	65.7
45–59	51.4	48.6		35.1	64.9
60+	48.6	51.4		31.1	68.9
Place of Residence					
Rural	37.4	62.6		40.5	59.6
Urban	50.1	49.9		26.6	73.4
Gender					
Male	44.0	56.0		34.4	65.6
Female	36.4	63.6		32.8	67.2
Marital Status					
Never Married/ divorced/	40.2	59.8		37.9	62.1
Currently married	40.1	59.9		29.0	71.0
Widowed	55.4	44.6		40.4	59.6
Social Groups					
ST	75.5	24.5		48.1	51.9
SC	53.4	46.6		22.5	77.5
OBC	34.4	65.6		47.9	52.1
General	42.8	57.1		20.4	79.6
Education					
Illiterate	52.0	48.0		44.8	55.2
Up to primary	14.2	85.8		39.1	60.9
Up to secondary	43.5	56.5		28.5	71.5
Above Secondary	53.7	46.3		21.5	78.5
Household occupation					
Self employed	41.8	58.2		30.3	69.7
Regular Wages	41.1	58.9		24.6	75.5
Casual Labourer	37.6	62.4		59.6	40.4
Economic quintile-Rural					
Poorest	34.4	65.6		48.6	51.4
Poor	33.1	66.9		23.0	77.0
Middle	44.1	55.9		33.6	66.4
Rich	49.3	50.7		22.2	77.8
Richest	40.5	59.5		39.4	60.7

Source: Authors’ computation from unit records of NSSO 75th Round 2017-18

Major reasons for non-utilization of public healthcare facilities during hospitalization were poor quality of available care or non-availability of doctors at public healthcare facilities (46.2%), preferences for the trusted provider (19.5%), non-availability of services (17.3%), and long waiting time (9.7%). See supplementary table A1 for reasons for not availing government healthcare facilities.

#### Outpatient care

The share of private facility utilization (66.1%) was considerably higher than the public facilities (33.9%) for outpatient care. Public facility utilization was higher among 0–14 years (53.1%), rural areas (40.5%), widowed (40.4%), ST category (48.1%), illiterate (44.8%), casual laborer (59.6%), and poorest income quintile (48.6%) compared to the respective counterparts. Private sector utilization was high among 60 and above age group, urban areas, currently married, general category, above secondary educated, regular wage household, and rich income quintile (Table 2).

Major reasons for not-availing services as public healthcare facilities in outpatient care were preference for the trusted doctor (49%), poor quality of available services (20.6%), non-availability of services at public healthcare facilities (20.6%), and long waiting time (11.1%). See supplementary table A1 for reasons for not availing government healthcare facilities.

### Financial protection: health insurance coverage, OOPE, and CHE-10

***Insurance coverage.*** Out of the total hospitalizations in the last 365 days, 23.5% of individuals had some insurance coverage (Table 3). A large share of this was publically funded health insurance coverage. Insurance coverage was higher among 60 years or above age group (35.0%), urban areas (29.1%), females (29.1%), SC category (27.4%), and rich income quintiles (41.5%; Table 3) compared to their respective counterparts.

**Table 3:** Health insurance coverage, out-of-pocket expenditure (OOPE), and catastrophic health expenditure (CHE-10) among individuals with mental disorders in India

	Insurance coverage (%) (n = 374)	OOPE during hospitalization in INR (n = 374)		OOPE during Out-patient care in INR (n = 283)		CHE-10 during hospitalization (%) (n = 304)	
		Public	Private	Public	Private	Public	Private
Total	23.5	7947	37,152	544	2358	30.8	82.5
Age group (years)							
0–14	12.8	6975	29,035	544	2463	30.8	82.4
15–29	27.8	8603	32,550	306	1051	41.0	71.1
30–44	23.9	10,712	35,166	378	1244	48.3	76.1
45–59	24.3	4881	37,330	650	1220	20.9	77.7
60+	35.0	6027	50,323	844	854	1.1	78.9
Place of Residence							
Rural	21.5	8162	30,314	420	3903	39.2	86.8
Urban	29.1	7631	46,839	752	980	10.7	67.0
Gender							
Male	21.5	8964	34,298	636	3047	33.5	82.4
Female	29.1	6067	41,539	366	1111	27.1	82.3
Marital Status							
Never Married/ divorced/	21.0	7673	27,403	387	4190	29.5	91.9
Currently married	22.7	8867	43,152	754	1189	32.6	74.8
Widowed	27.2	4017	21,968	438	890	27.0	52.5
Social Groups							
ST	18.5	5039	33,854	201	399	28.6	80.8
SC	27.4	6901	32,825	1094	930	28.2	63.1
OBC	23.6	6616	40,345	343	4437	32.9	85.3
General	21.9	11,293	35,433	970	1350	30.1	83.7
Education							
Illiterate	27.4	6551	24,497	427	1302	30.4	91.1
Up to primary	4.8	6742	57,849	372	5526	30.3	90.5
Up to secondary	21.6	9557	30,249	586	1088	16.8	64.9
Above Secondary	39.2	9910	45,533	1082	1347	55.9	81.1
Household occupation							
Self-employed	15.4	10,273	45,791	456	3956	34.8	84.6
Regular Wages	29.3	4777	25,744	642	560	19.6	70.6
Casual Labourer	25.1	5981	26,760	430	1603	39.9	85.5
Economic quintile							
Poorest	3.4	12,798	59,502	609	1094	40.7	95.7
Poor	14.6	5837	38,767	709	815	55.7	94.9
Middle	21.1	3785	28,316	757	6405	3.6	81.6
Rich	41.5	6280	26,633	234	1098	23.0	74.3
Richest	35.7	9591	35,036	445	1483	37.5	58.1

Source: Authors’ computation from unit records of NSSO 75th Round 2017-18

#### Hospitalization-associated OOPE

Average OOPE was 7,947 INR (123 USD) under public facilities and 37,152 INR (576 USD) under private facilities in India (Table 3). OOPE under private facilities was substantially higher compared to public facilities. For example, OOPE for hospitalization in the age group 60 years and above was 6,027 INR (94 USD) in public facilities and 50,323 INR (781 USD) under private facilities. Similarly, OOPE in urban areas was 7,631 INR (118 USD) under public facilities and 46,839 INR (727 USD) under private facilities (Table 3).

A major source of financing hospitalization expenses was household income or savings (75.5%). However, one in four individuals had to borrow money to meet the hospitalization expenses. See Supplementary Table A2 for sources of financing hospitalization expenses.

#### Out-patient care-associated OOPE

Average OOPE for an out-patient visit was 544 INR (8 USD) under public facilities and 2,358 INR (37 USD) under private facilities. Under public facilities, OOPE was high in urban areas [752 INR (12 USD)], for males [636 INR (10 USD)], and those currently married [754 INR (12 USD)], or have above secondary education [1,082 INR (17 USD)], and are regular wage households [642 INR (10 USD)] compared to their respective counterparts. OOPE under the private sector was higher than in the public sector and more so in the 0–14 years age group [2,463 INR (38 USD)], rural areas [3,903 INR (61 USD)], males [3,047 INR (47 USD)], and self-employed [3,956 INR (61 USD)] households. Table 3.

#### CHE-10

On average, about 30.8% of individuals with mental disorders reported CHE-10 due to hospitalization under public facilities, and about 82.5% reported CHE-10 under private facility hospitalization (Table 3). CHE-10 was considerably high in the lower socioeconomic population group. For example, 95.7% of the poorest income quintile households with hospitalization under the private sector faced CHE-10 in the last 365 days (Table 3).

### Medical and non-medical expenditures during hospitalization and outpatient care

***Hospitalization associated expenditures***: Total expenditure during a hospitalization was 8,794 INR (medical expenditure: 5,932 INR [92 USD], non-medical expenditure: 2,862 INR [44 USD]) under public facilities and 30,331 INR (medical expenditure: 27,294 INR [423 USD], non-medical expenditure: 3,035 INR [47 USD]) under private facilities. The share of average medical expenditure of the total expenditure was 67.5% under public facilities, and 90.0% under private facilities. The average expenditure on medicines was 3,958 INR (61 USD) under public facilities and 11,987 INR (186 USD) under private facilities, which were 45.0% and 39.5% of total expenditure, respectively. Bed charges had a share of 16.2% of total expenditure under private facilities and 1.9% under public facilities (Table 4).

**Table 4:** Cost of medical and non-medical expenditure during inpatient and outpatient care for mental disorders in India

Strata	Average medical expenditure in INR and %						Average non-medical expenditure in INR and %			Total
	Doctor/ Surgeon Fee	Medicine	Diagnostic Test	Bed Charge	Other medical expense	Total	Transport for patient	Other non-medical expense	Total	
**Inpatient care (n = 374)**										
Public INR	54	3958	1199	164	557	***5932***	1200	1662	***2862***	8794
%	0.6	45	13.6	1.9	6.3	***67.5***	13.6	18.9	***32.5***	100
Private INR	4423	11,987	3687	4923	2273	***27,294***	1100	1935	***3035***	30,331
%	14.6	39.5	12.2	16.2	7.5	***90***	3.6	6.4	***10***	100
**Outpatient care (n = 283)**										
Public INR	2	438	19	-	22	***482***	62	47	***109***	591
%	0.3	74.1	3.2		3.7	***81.6***	10.5	8.0	***18.4***	100
Private INR	169	1091	380	-	790	***2430***	183	138	***321***	2751
%	6.1	39.7	13.8		28.7	***88.3***	6.7	5.0	***11.7***	100

Source: Authors’ computation from unit records of NSSO 71st, 2014, and 75th Round 2017-18

***Outpatient care-associated expenditures.*** Total expenditure per outpatient visit under public facilities was 591 INR (medical expenditure: 482 INR [7 USD], non-medical expenditure: 109 INR [2 USD]) and 2,751 INR under private healthcare facilities (medical expenditure: 2,430 INR [38 USD], non-medical expenditure: 321 INR [5 USD]). Expenditure on medicines was 438 INR (7 USD) under public facilities and 1,091 INR (17 USD) under private facilities, which constituted 74.1% and 39.7% of total expenditure, respectively. (Table 4)

### Predictors of access (incidence of hospitalization and outpatient visit) and financial protection (CHE-10)

***Hospitalization***: The hospitalization rate due to mental disorders was 20 per 1,00,000 individuals in India (Table 1). The chance of hospitalization increased with the increasing age group. For instance, the chance of hospitalization in the age group 60 years and above was 4.19 times higher [(95% CI: 2.71–6.48); *p* < 0.001] compared to the 0–14 years age group. The chance of hospitalization was also higher in the rural areas, male, and general category population compared to their counterparts and this was statistically significant. Insurance coverage did not affect the chance of hospitalization (Table 5).

**Table 5:** Factors predicting hospitalization, proportion of ailing population (PAP), and catastrophic health expenditure (CHE-10) due to mental disorders in India, 2017-18

Total	Reporting of hospitalization OR (95% CI)	Reporting of PAP OR (95% CI)	Reporting of CHE-10 OR (95% CI)
**Age group (years, ref:0–14)**			
15–29	2.48 (1.69–3.64)*	NA	1.22 (0.34–4.34)
30–44	3.44 (2.35–5.03)*	NA	0.49 (0.10–2.34)
45–59	4.13 (2.81–6.08)*	NA	0.24 (0.05–1.24)
60+	4.19 (2.71–6.48)*	NA	0.15 (0.03–0.87)**
**Place of Residence (ref: rural)**			
Urban	0.77 (0.61–0.98)**	0.96 (0.74–1.24)	0.38 (0.18–0.80)**
**Gender (ref: male)**			
Female	0.62 (0.50–0.77)*	0.82 (0.65–1.04)	0.56 (0.28–1.11)
**Marital status (ref: never married)**			
Currently married	NA	NA	3.73 (1.15–12.04)**
Widowed	NA	NA	1.10 (0.19–6.14)
**Social Groups (ref: ST)**			
SC	2.13 (1.32–3.43)**		2.41 (0.63–9.11)
OBC	2.17 (1.41–3.36)*	2.95 (1.51–5.79)**	1.78 (0.53–5.98)
General	2.16 (1.38–3.38)**	3.73 (2.01–6.93)*	2.48 (0.71–8.67)
**Education (ref: illiterate)**			
Up to primary	NA	NA	0.42 (0.15–1.23)
Up to secondary	NA	NA	0.52 (0.21–1.26)
Above Secondary	NA	NA	1.18 (0.42–3.32)
**Household occupation (ref: self-employed)**			
Regular Wages	1.24 (0.73–1.42)	1.03(0.76–1.40)	0.54 (0.23–1.30)
Casual Labourer	0.88 (0.65–1.19)	0.84 (0.59–1.19)	1.29 (0.54–3.10)
**Economic quintile (ref: poorest)**			
Poor	1.02 (0.73–1.42)	1.00 (0.67–1.50)	0.99 (0.33–3.02)
Middle	0.89 (0.64–1.25)	1.33 (0.91–1.93)	0.33 (0.11–0.98)
Rich	1.05 (0.75–1.46)	1.25 (0.84–1.84)	0.24 (0.09–0.66)**
Richest	1.13 (0.80–1.58)	1.73 (1.18–2.52)**	0.29 (0.10–0.82)**
**Insurance coverage (ref: No)**			
Yes	1.11 (0.85–1.44)	NA	0.43 (0.18–1.03)
**Provider (ref: public)**			
Private	NA	NA	23.33(10.85–50.17)*
**Constant**	0.00 (0.00–0.00)*	0.00 (0.00–0.00)**	0.75 (0.14–3.96)
**Model Details**			
Log likelihood	-2932.89	-2392.59	-129.00
Number of observations	555,351	555,351	292
LR Chi2	139.70	71.99	143.71
Prob > Chi2	0.000	0.000	0.000
Pseudo R2	0.023	0.014	0.357
Mean Variance inflation factor	1.44	1.49	2.38
Mean Pregibon dbeta	0.001	0.001	0.55
Specification error (linktest): predicted value (_hat)[p>|z|]	0.01	0.04	0.000
Specification error (linktest): predicted value squared (_hatsq) [p>|z|]	0.07	0.19	0.990

Note: (*) *p*-value < 0.001; (**) *p*-value < 0.05;‘NA’ indicates particular variable was not included the respective model.

All estimates, except model details, are odds ratio (OR) and values in the parentheses are confidence intervals of the estimates

Source: Authors’ computation from unit records of NSSO 75th Round 2017-18

***Acute/chronic illness***: The proportion of ailing population (PAP) due to mental disorders in the last 15 days was 40 per 1,00,000. Self-reporting of ailment was higher in the general category [OR: 3.73; 95% CI: 2.01–6.93; *p* < 0.001] compared to ST category, and richest income quintile [OR: 1.73; 95% CI: 1.18–2.52; *p* < 0.001] compared to poorest income quintile (Table 5).

***CHE-10***: The chance of facing CHE-10 was 62% lower [95% CI: 82 − 20; *p* < 0.05] in urban areas compared to rural areas. The chance of facing CHE-10 was 23.33 times higher [95% CI: (10.85–50.17); *p* < 0.001] under the private sector compared to the public sector (Table 5).

## Discussion

The current study aimed to assess progress towards UHC among individuals with mental disorders by measuring the gap in access and financial protection in India. Utilization has been used as one of the proxy indicators of access. In addition to providing a descriptive summary of characteristics associated with public and private sector hospitalization and outpatient care use (access indicators), we examined characteristics of health insurance coverage, OOPE, and CHE-10 (financial risk protection indicators) among individuals with mental disorders. Most importantly, we identified predictors of access and financial protection. We used the 75th NSS, which is nationally representative and one of the most robust datasets present in the country [15]. Findings of the study have been discussed under the following two headings: (1) Service utilization (access to healthcare), and (2) Financial Protection.

### Service utilization (access to healthcare)

The current study showed that the private sector was a major service provider for mental health services with a larger share for outpatient than inpatient care. Since inpatient care requires greater investment in infrastructure, human resources, clinical management and legal aspect than outpatient care, the private sector invest less in inpatient care than outpatient care [24]. After the Mental Healthcare Act 2017, mental health establishment comes with new legal and healthcare aspects unlike other health conditions. Interestingly, in individuals from higher socioeconomic groups, there was lower utilization of private healthcare facilities during inpatient care compared to poorer socioeconomic groups. A potential explanation could be that individuals from lower socioeconomic groups may not have access to government mental health hospitals, typically situated in few metropolitan cities in India, for inpatient care whereas those from higher socioeconomic groups may have greater access to government facilities since they can afford higher medical expenditure including transportation costs [26, 35]. This pattern of utilization could also be due to regional and state variations. In recent years, given the rise in mental health awareness, the private sector has also started investing in mental health in urban localities [25]. Public healthcare utilization was higher among ST group compared to other caste group in India and similar findings were also reported by previous studies [15, 19]. Study done by Indian Council of Medical Research (ICMR) on urban mental health shows that higher proportion of poor population depend on public sector for access to inpatient care, whereas for outpatient care informal private sector was significant service provider [26]. However, further research is required to understand the differences in patterns of health care utilization among different socioeconomic groups.

In the present study, 63.5% of the individuals with mental disorders who went to the private sector for hospitalization reported unavailability or poor service quality at public facilities. While tax-funded government health facilities provide selective care related to immunization, maternal and child health, leprosy, etc., there are limited resources for mental disorders [27, 28]. For India’s population of 1.39 billion, there are 9,000 psychiatrists, 2,000 psychiatric nurses, 1,000 psychiatric social workers, 1,000 clinical psychologists, and 60,000 psychiatric beds [29, 30]. In other words, India has 0.3 psychiatrists per 100,000 population compared to the global median of 3 per 100,000 population. Canada and New Zealand recommend a range of one psychiatrist for 7,500 to 10,000 population [31, 32]. This gap between the demand for mental health services and their supply has created significant unmet healthcare needs [33].

In outpatient care, preference for a trusted doctor was the major reason for choosing a private provider over a public provider. This was mainly due to greater autonomy to visit the same doctor for continuity of care under the private sector than the public sector. Patients often find it difficult to consult the same doctor under public facilities during their follow-up visits. Similar findings have also been reported by various national and international studies [34, 35].

In light of findings of the present and previous studies, there is an urgent need for the government to involve the private sector in care provisioning. Recent report of National Human Rights Commission (NHRC), India states deplorable conditions of all 46 Government Mental Healthcare Institutions in the country [36]. Even with an abysmal psychiatrist – population ratio, 75% of the psychiatrists in India work under the private sector to provide inpatient and outpatient services [24, 37]. In the last few decades’ non-governmental organization like Sangath Society, Goa, Schizophrenia Research Foundation, Chennai, and Medico-Pastoral Association, Bangalore, have contributed meaningfully to address mental health needs of the country [38]. They provided a wide range of services for conditions including child mental health, schizophrenia and psychotic conditions, drug and alcohol abuse and dementia.

### Financial risk protection

Financial hardship was a major challenge in access to mental health services in India. Our analyses showed that more than three out of four hospitalized individuals did not have health insurance coverage, and one out of four had to borrow money for hospitalization. Access to services becomes even more challenging since outpatient care and rehabilitation services are not covered under most health insurance schemes in India [39, 40]. Individuals with mental disorders often require life-long services and expenditure on drugs and diagnostics constitutes more than half of the total expenditure. Previous studies have also shown a similarly high level of financial hardship in outpatient care compared to inpatient care over time [41]. Lack of health insurance coverage leads to higher OOPE at the point of service delivery for the household, which is one of the most regressive financing methods. Coverage of health insurance becomes more important for individuals with mental disorders in the lower socioeconomic population group since their disease burden is higher compared to richer population group [7, 8, 37]. International research shows that health insurance is more sensitive towards outcome of mental illness than physical illness [42]. India’s Mental Healthcare Act, 2017 also places mental illness at par with physical health and directs health insurance companies to cover mental health [43]. In this context, Ayushman Bharat Scheme- Pradhan Mantri-Jan Arogya Yojana, India’s flagship health program, also covers mental disorders in its service packages. However, most of the health insurance schemes in India predominantly cover hospitalization and do not cover (or minimally cover) outpatient care, follow-up visits, rehabilitative, and long term care [44, 45]. Given the high utilization of the private sector, there is a critical need for health insurance to cover private sector mental health care services.

OOPE was many times higher under the private sector compared to the public sector in India with more than eight out of 10 households facing CHE-10 during hospitalization. This could be attributed to the profit maximization nature of India’s private sector and differential charging schemes wherein patients are charged till their maximum capacity to pay [28, 35]. In contrast to private facilities, public facilities appear to be more equitable given that their outpatient utilization among the lower socioeconomic groups was higher than higher socioeconomic groups. However, OOPE for drugs and diagnostics under the public sector is still significant, which may reduce affordability for patients from lower socioeconomic groups.

Often, individuals with a mental health disorder from lower socioeconomic groups and rural areas may either forgo their treatment or turn to traditional healers or informal providers that are not legally approved in the country [46]. This could be due to the unavailability of mental health facilities at the primary healthcare facilities (health sub-centre, primary health centre, and community health centre), which are closest to the community [29, 47]. Moreover, the private sector does not typically invest in setting up mental health facilities in remote and rural areas of India [37]. For individuals from lower socioeconomic groups, even non-medical expenditure (transportation, food, lodging) can hinder access to care, apart from opportunity cost [48, 49]. Taken together, a variety of factors contribute to the high unmet healthcare needs for mental disorders in India [33, 50].

India’s health system has been chronically underfunded since the government allocates only 1.3% of its gross domestic product (GDP), which is much less than the 5% recommended by the World Health Organization [51, 52]. India’s National Health Policy had proposed spending 2.5% of the GDP on health by 2025, but this commitment has not been realized so far [52]. Among the competing healthcare priorities, the country has paid very little attention to mental health. India spends less than 1% of total government health expenditure on mental health (mental health hospital: 0.23%; rehabilitative care: 0.05%; all long term care: <0.01%) [51]. In the latest union budget for financial year 2023-24, INR 89,155 crores were allocated to the Ministry of Health and Family Welfare (MoHFW) for health; out of that, INR 919 crores, which is 1.03% of the budget estimates of MoHFW, was allocated for mental health [53]. Other than direct allocation from the health department, INR 280 crores were allocated from the Ministry of Social Justice and Empowerment (MoSJE). Total budget estimates (MoHFW and MoSJE) for mental health in the financial year 2023-24 was INR 1,199 crores. Of the total INR 919 crores (direct expenditure on mental health by MoHFW), 85% funds just two institutes in the country -(i) National Institute of Mental Health and Neuro-Sciences, (NIMHANS), Bengaluru (INR 721 crore); and (ii) Lokpriya Gopinath Bordoloi Regional Institute of Mental Health, Tezpur (INR 64 crore). In addition, INR 134 crores funds the National Tele-Mental Health Programme, announced in October 2022. The overall budget for the National Mental Health Programme (NMHP), which came under the line item of tertiary activities of the National Health Mission, has dropped by 42% from INR 500 crores in financial year 2022-23 to INR 290 crores in 2023-24 [53]. This reduction in NMHP financing is a matter of concern since it funds tertiary-level institutions in the country. This is particularly concerning in light of the National Human Right Commission’s report on the poor condition of government mental health institutions and the acute shortage of mental health professionals [36]. According to one conservative estimate, the annual estimated cost for the government to meet the mental health needs of the country would be INR 94,073 crores [29].

The current COVID-19 pandemic is a wake-up call for greater investment in mental healthcare in India. Issues related to mental health have been reported across the world and in India [54]. The National Health Policy-2017 envisages providing comprehensive primary healthcare, including mental health, at the community level by upgrading the health sub-centre to a health and wellness centre. However, real-world implementation has yet to be realized [55]. As the country is going through an epidemiological and demographic transition, by 2050, 20% of India’s population will be above the age of 60 years. The large elderly population may present with a higher disease burden of non-communicable diseases and mental health disorders [56]. Thus, there is an urgent need to improve the country’s mental health resources.

The findings of this study need to be interpreted in light of certain limitations. First, NSS is a self-reported survey that might miss individuals’ actual healthcare needs, since self-reporting depends on the sociodemographic characteristics of the respondent [57]. This point have been highlighted by previous studies and Longitudinal Ageing Study of India (LASI) [33, 58]. In comparison to 75th NSS, mental health was comprehensively captured under NMHS, 2015-16. Second, the 75th NSS data is reliable for mental disorders at the national level but not at the state level due to inadequate sample size. Hence, regional variation in the estimates were not provided. Second, this study uses utilization as a proxy indicator of access, but access is a multidimensional concept which cannot be equated with utilization [33, 59]. Merely being treated by a healthcare provider is not adequate access. Health needs are also unmet if the health care provided is inappropriate. The Tanahashi framework refers to this as care that is not effective [59], and so does the UHC definition. Also, the 75th NSS combined different categories of mental disorders into one category that does not allow a deeper understanding of individual mental disorders, which must be calculated for populous countries like India. However, the study’s strengths include the nationally-representative sample which allows for investigation into factors affecting healthcare utilization and financial protection in the present context.

## Conclusion

There remain significant gaps in access and financial protection among individuals with mental disorders in India. The private sector is a major service provider for individuals with mental disorders with a greater share of outpatient care compared to inpatient care. However, financial hardship is considerably very high while seeking care from the private sector. The public sector is more affordable and equitable compared to the private sector. However, the public sector provides a limited range of services that may not meet the societal demand for mental health services. India needs greater investment in mental health resources as it goes through an epidemiological and demographic transition. To achieve UHC the country needs to strengthen its healthcare system and urgently address the gaps in access and financial risk protection.

### Electronic supplementary material

Below is the link to the electronic supplementary material.

Supplementary Material 1

## Data Availability

The present study is based on India’s National Sample Survey, 2017-18, which is freely available in the public domain (http://www.mospi.gov.in/unit-level-data-report-nss-75th-round-july-2017-june-2018-schedule-250social-consumption-health) [15].

## List of abbreviations

CHC: Community Health Centre
CHE: Catastrophic health expenditure
CHE-10: Proportion of households in a population who face catastrophic health expenditure computed using the threshold of 10% of usual annual consumption expenditure
DH: District Hospital
NSS: National sample survey
NSSO: National sample survey office
OOPE: Out-of-pocket expenditure
PHC: Primary Health Centre
PMJAY: Pradhan Mantri Jan Arogya Yojana
SHC: Sub Health Centre
RSBY: Rashtriya Swasthya Bima Yojana
