# Supplementary material for: Progress towards universal health coverage in the context of mental disorders in India: evidence from national sample survey data
Source: Int J Ment Health Syst. 2023 Sep 19;17:27. doi: 10.1186/s13033-023-00595-6 (PMC10507945; doi:10.1186/s13033-023-00595-6)
Supplement: Supplementary file 1 — Supplementary Material 1 [file 13033_2023_595_MOESM1_ESM.docx]

| Table A1: Reasons for not availing government healthcare facilities during hospitalization and outpatient care among individuals with mental disorders in India, 2017-18. | | | | | | | |
| --- | --- | --- | --- | --- | --- | --- | --- |
|  | Service not available | Available but poor quality /doctor not available | Quality satisfactory but too far | Quality satisfactory but long waiting time | Financial constraints | Preference for trusted doctor /hospital | Others |
| Hospitalization (n=203) | |  |  |  |  |  |  |
| Total | 17.3 | 46.2 | 2.8 | 9.7 | 0.3 | 19.5 | 3.3 |
| Place of Residence |  |  |  |  |  |  |  |
| Rural | 14.9 | 50.1 | 3.9 | 7.1 | 0.4 | 20.3 | 3.3 |
| Urban | 25.6 | 32.6 | 3.4 | 18.6 | 0.0 | 16.8 | 3.0 |
| Sex |  |  |  |  |  |  |  |
| Male | 23.2 | 30.6 | 6.2 | 11.8 | 0.0 | 23.5 | 4.7 |
| Female | 10.4 | 64.6 | 0.9 | 7.1 | 0.6 | 14.7 | 1.6 |
| Economic class |  |  |  |  |  |  |  |
| Poorest | 16.0 | 76.1 | 0.8 | 2.7 | 0.0 | 2.9 | 1.4 |
| Richest | 19.0 | 31.4 | 8.1 | 14.2 | 1.5 | 16.1 | 9.9 |
| Outpatient visit (n=147) | |  |  |  |  |  |  |
| Total | 12.7 | 20.6 | 6.2 | 11.1 |  | 49.0 | 0.4 |
| Place of Residence |  |  |  |  |  |  |  |
| Rural | 24.4 | 28.3 | 13.0 | 7.3 |  | 26.2 | 0.8 |
| Urban | 2.3 | 13.8 | 0.1 | 14.4 |  | 69.4 | 0.1 |
| Sex |  |  |  |  |  |  |  |
| Male | 16.6 | 13.0 | 2.5 | 11.0 |  | 56.2 | 0.6 |
| Female | 5.7 | 34.4 | 12.8 | 11.1 |  | 36.0 | 0.0 |
| Economic class |  |  |  |  |  |  |  |
| Poorest | 6.6 | 53.3 | 0.0 | 1.1 |  | 35.9 | 3.1 |
| Richest | 21.4 | 9.6 | 4.0 | 23.5 |  | 41.3 | 0.1 |
| Source: Authors’ computation from unit records of NSSO 75^th^ Round 2017-18 | | | | | | | |

| Table A2: Source of financing for hospitalization expenses among individuals with mental disorders. | | | | | |
| --- | --- | --- | --- | --- | --- |
|  | Household income/saving | Borrowing | Sale of physical asset | Contribution from friends and relatives | Other sources |
| Hospitalization (n=374) |  |  |  |  |  |
| Total | 75.5 | 12.0 | 0.6 | 9.4 | 2.5 |
| Place of Residence |  |  |  |  |  |
| Rural | 75.1 | 12.1 | 0.8 | 9.9 | 2.1 |
| Urban | 76.4 | 12.0 | 0.0 | 7.9 | 3.7 |
| Sex |  |  |  |  |  |
| Male | 72.2 | 13.7 | 0.4 | 11.7 | 2.0 |
| Female | 79.9 | 9.8 | 0.8 | 6.3 | 3.1 |
| Outpatient visit (n=283) |  |  |  |  |  |
| Total | 95.9 | 0.4 |  | 1.1 | 2.7 |
| Place of Residence |  |  |  |  |  |
| Rural | 93.6 | 0.3 |  | 2.1 | 4.1 |
| Urban | 98.4 | 0.4 |  | 0.0 | 1.1 |
| Sex |  |  |  |  |  |
| Male | 97.5 | 0.0 |  | 0.7 | 1.8 |
| Female | 92.4 | 1.2 |  | 2.0 | 4.5 |
| Source: Authors’ computation from unit records of NSSO 75^th^ Round 2017-18 | | | | | |
